# Excreted Thiocyanate Detects Live Reef Fishes Illegally Collected Using Cyanide—A Non-Invasive and Non-Destructive Testing Approach

**DOI:** 10.1371/journal.pone.0035355

**Published:** 2012-04-20

**Authors:** Marcela C. M. Vaz, Teresa A. P. Rocha-Santos, Rui J. M. Rocha, Isabel Lopes, Ruth Pereira, Armando C. Duarte, Peter J. Rubec, Ricardo Calado

**Affiliations:** 1 Departamento de Biologia & CESAM, Universidade de Aveiro, Campus Universitário de Santiago, Aveiro, Portugal; 2 Departamento de Química & CESAM, Universidade de Aveiro, Campus Universitário de Santiago, Aveiro, Portugal; 3 ISEIT/Viseu, Instituto Piaget, Galifonge, Lordosa, Viseu, Portugal; 4 International Marinelife Alliance, Saint Petersburg, Florida, United States of America; Leibniz Center for Tropical Marine Ecology, Germany

## Abstract

Cyanide fishing is a method employed to capture marine fish alive on coral reefs. They are shipped to markets for human consumption in Southeast Asia, as well as to supply the marine aquarium trade worldwide. Although several techniques can be used to detect cyanide in reef fish, there is still no testing method that can be used to survey the whole supply chain. Most methods for cyanide detection are time-consuming and require the sacrifice of the sampled fish. Thiocyanate anion (SCN*^−^*) is a metabolite produced by the main metabolic pathway for cyanide anion (CN^−^) detoxification. Our study employed an optical fiber (OF) methodology (analytical time <6 min) to detect SCN*^−^* in a non-invasive and non-destructive manner. Our OF methodology is able to detect trace levels (>3.16 µg L^−1^) of SCN^−^ in seawater. Given that marine fish exposed to cyanide excrete SCN*^−^* in the urine, elevated levels of SCN*^−^* present in the seawater holding live reef fish indicate that the surveyed specimens were likely exposed to cyanide. In our study, captive-bred clownfish (*Amphiprion clarkii*) pulse exposed for 60 s to either 12.5 or 25 mg L^−1^ of CN^−^ excreted up to 6.96±0.03 and 9.84±0.03 µg L^−1^ of SCN^−^, respectively, during the 28 days following exposure. No detectable levels of SCN^−^ were recorded in the water holding control organisms not exposed to CN^−^, or in synthetic seawater lacking fish. While further research is necessary, our methodology can allow a rapid detection of SCN^−^ in the holding water and can be used as a screening tool to indicate if live reef fish were collected with cyanide.

## Introduction

Coral reefs currently face an increasing number of anthropogenic threats worldwide (e.g., coastal development, agricultural runoff, and overfishing) [1]–[4] which are magnified by global climate change (e.g. coral bleaching induced by increasing water temperatures and ocean acidification due to increasing carbon dioxide emissions) [5], [6].

Coral reefs in Indonesia and the Philippines are currently the most at risk (about 95% of existing reefs), due to the use of destructive fishing techniques [7]. Millions of fish are collected illegally from coral reefs every year using cyanide, with over 500 metric tons of cyanide being used annually for this practice on Philippine reefs alone [8]–[10]. While highly priced groupers are shipped live for human consumption to Hong Kong and other Asian countries [9], [11], other marine fish are transported mainly to the USA, the European Union, and Japan to supply the marine aquarium trade [12].

Cyanide fishing is an inexpensive, highly-effective method used to stun fish as follows: 1) fishermen insert sodium cyanide (NaCN) or potassium cyanide (KCN) tablets into squirt bottles containing seawater; 2) they squirt hydrogen cyanide (HCN) solution into coral crevices sheltering the fish; 3) the cyanide temporarily stuns some of the fish (many die from acute doses); and 4) stunned fish are brought to fishing boats and put into fresh seawater to recover [13]. Cyanide is extremely destructive to marine organisms, killing both targeted and non-targeted specimens [14]. Despite leaving the reef physically intact (although corals may be broken to extract stunned fish), cyanide kills coral polyps by disrupting their symbiotic association with the zooxanthellae [15], [16]. Although cyanide fishing is illegal in most exporting countries, it is still a common practice, and is often encouraged by corrupt authorities taking advantage of poor rural communities [17].

Cyanide-ion selective electrodes (ISE) have been successfully used to identify live reef fish collected with cyanide up to 14 days post-exposure [18], although other methods could potentially be used (e.g. titrimetric, colorimetric, GC-MS, enzyme-based biosensors, or biomarker approaches) to detect cyanide or cyanide metabolites, after fish tissues have been digested to release the cyanide into solution [19]–[22]. The concentrations of cyanide (or its metabolites) present in live fish tissues, blood, or urine may vary by fish size and species, as well as in relation to: 1) the concentration of the cyanide solution used during their capture; 2) the duration of the exposure period; 3) post-collection handling; 4) the holding time in exporting or importing facilities; and 5) shipping duration [23]. The current gaps in knowledge of the pharmacokinetics of cyanide and its major metabolites in marine fish impairs researchers' ability to identify the most appropriate cyanide testing methods that could be applied along the supply chain [23]. Live reef fishes are highly priced goods. Therefore, any method requiring their sacrifice to detect cyanide would not be welcome by most exporters, importers, or retailers. Consequently, it is preferable to find a reliable method using a non-invasive and non-destructive approach to determine whether live reef fishes were collected with cyanide.

The major pathway for cyanide metabolism is the conversion of cyanide (CN^−^) to thiocyanate (SCN^−^), in the presence of a sulfur donor, by the enzyme rhodanese (thiosulfate sulfurtransferase; EC 2.8.1.1) [24]. This mitochondrial enzyme has been isolated from fish gills and intestine, and it is most active in the liver and kidneys [25], [26]. Nearly 80% of all cyanide entering the organism is converted to SCN^−^ and later excreted in the urine [21]. Although this detoxification process has already been documented in freshwater fishes, available information is based mostly on chronic cyanide exposure trials, rather than acute pulse dosage using high cyanide concentrations over short time intervals (e.g. 30–90 s) similar to those employed to collect reef fish with cyanide [23], [27]–[30]. Additionally, freshwater fish display a dramatically different strategy from marine fish in the maintenance of their osmotic balance. Freshwater fish have a high rate of urinary excretion, while marine fish have a low urinary excretion rate because they must retain water in their blood to maintain osmotic balance with the surrounding seawater [31], [32].

The present study tests the following hypothesis: if marine fish can retain SCN^−^ for long periods following their exposure to cyanide [18], [33], the excretion of SCN^−^ may be used to detect live reef fishes collected illegally with cyanide several weeks after their capture. This hypothesis will be tested by employing a sensor capable of detecting SCN^−^ concentrations in water used to hold/ship these organisms. The rationale is that fish collected illegally with cyanide will excrete urine containing SCN^−^ into the seawater of holding tanks or plastic shipping bags employed during their export. Elevated levels of SCN^−^ present in seawater can be detected by employing an optical fiber sensor capable of measuring trace aqueous concentrations of SCN^−^ [34].

The present study sought to determine: 1) if abnormal levels of SCN^−^ can be detected noninvasively and nondestructively, by testing the seawater used during the shipping/holding of live reef fish; 2) how long after pulse exposure to cyanide do reef fish start to excrete SCN^−^ in detectable levels; and 3) whether fish continue to excrete SCN^−^ in detectable levels at least 4 weeks after their pulse exposure to CN^−^.

## Materials and Methods

### Selection of the fish model species and husbandry

Nearly half of all live reef fish traded by the marine aquarium industry belong to the family Pomacentridae (damselfish) [12]. The use of cyanide to collect damselfish has already been reported [18], thus it was reasonable to select a species from this family as a model for the present work. In order to assure that selected specimens had never been previously exposed to cyanide, two possible options were available: 1) select a model species whose specimens could be collected from the wild, namely from regions with no previous records of cyanide fishing; or 2) select a model species whose specimens could be cultured in captivity and thus assure that they had never been exposed to cyanide. As traceability in the trade of live reef fish is far from being reliable [35], the first option was rejected and the use of cultured specimens in captivity was considered the most suitable approach for the present work. Members of the genus *Amphiprion* (one of the two genera of clownfish) are some of the most popular species among marine aquarium hobbyists [12] and, compared to other damselfishes, can be easily cultured in captivity [35], [36]. Recently, local marine aquarium retailers have reported the occurrence of high mortalities in imports of wild clownfish, namely Clark's clownfish *Amphiprion clarkii* (Bennett, 1830), and have attributed these deaths to cyanide poisoning. Although these reports are mostly anecdotal, it was decided to select *A. clarkii* as the model fish species for the present study.

Twenty-seven cultured specimens of *A. clarkii* with an average total length (measured from the tip of the snout to the tip of the longer lobe of the caudal fin) of 40.3± standard error (SE) 3.1 mm, and an average wet weight (± SE) of 1.8±0.2 g were purchased from a local producer (Opérculo Lda., Portugal), thus assuring that all specimens to be used in this work had never been exposed to cyanide. All specimens were kept for 60 days to acclimate to laboratory conditions in a 270-L glass tank (1.20 m long, 0.45 m wide and 0.50 m high) equipped with an internal circulation pump (Turbelle® nanostream - 6025, Tunze®, 2500 L h^−1^) and a metal halide lamp (150 W, 10,000 K, Sylvania®) providing a 12 h light:12 h dark photoperiod. The glass tank was connected to a 125-L sump (1.20 m long×0.35 m wide×0.40 m tall) equipped with a recirculating water pump (Eheim® 1262, 3400 L h^−1^), a 50 µm mesh bag for mechanical filtration, a Deltec® APF600 protein skimmer, a biological filter of submerged plastic bio-balls and a 300 watt submersible heater (Eheim® Jäger) keeping water temperatures stable at 26±0.5°C. This system was also equipped with an osmoregulator (Reef Set®) used to regulate the water level by replacing evaporated water with freshwater purified by reverse osmosis to keep salinity at 35 g L^−1^ (salinity was checked daily using a hand refractometer to detect any potential malfunction of the described automation). The fish maintenance system used seawater prepared by mixing RO water with a synthetic salt mix (Tropic Marine® Pro Reef salt). Water quality parameters were monitored every other day using colorimetric tests (Salifert®) with the exception of pH, which was monitored using a Pinpoint® pH meter (PH 370 by American Marine) and remained within optimal ranges for *A. clarkii* (pH 8.1±0.1; ammonium and nitrite not detectable, nitrate below 5 mg L^−1^). All fish were fed daily to satiation with a commercial pelleted food (Hikari® Marine S).

### Cyanide pulse exposure and depuration

**Figure 1:**
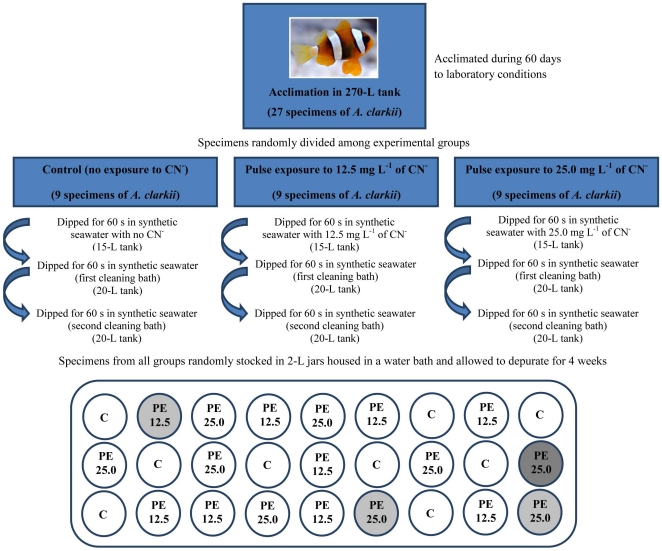
Schematic representation of experimental procedures for cyanide (CN^−^) pulse exposure and depuration of *Amphiprion clarkii*. C – control specimens not exposed to CN^−^; PE 12.5 – Specimens pulse exposed during 60 s to 12.5 mg L^−1^ of CN^−^; PE 25.0 - Specimens pulse exposed during 60 s to 25.0 mg L^−1^ of CN^−^; Circles with a white background represent specimens surviving until the end of the depuration period, while light and dark grey backgrounds represent specimens dying at least 30 min after the pulse exposure and during the depuration period, respectively.

After the acclimation period (60 days), the 27 *A. clarkii* were randomly divided into 3 groups of 9 fish for cyanide pulse exposure. The first group was used as a control (no exposure to cyanide), while the second group was exposed to a nominal concentration of 12.5 mg L^−1^ of CN^−^, and the third group was exposed to a nominal concentration of 25.0 mg L^−1^ of CN^−^. The pulse exposure to CN^−^ was performed in three steps: 1) exposure bath; 2) first cleaning bath; and 3) second cleaning bath. The exposure bath was performed as follows: all fish from the same group were collected with a hand-net and dipped for 60 s into a 15-L tank filled with: a) synthetic seawater with no cyanide (group 1); b) synthetic seawater dosed with CN^−^ at a concentration of 12.5 mg L^−1^ (group 2); and c) synthetic seawater dosed with CN^−^ at a concentration of 25.0 mg L^−1^ (group 3). After the pulse exposure to cyanide all fish from the same group were dipped for 60 s into a 20-L tank filled with synthetic seawater with no cyanide (first cleaning bath). This procedure was repeated one more time for all fish from all groups (second cleaning bath; see Figure 1 for a schematic representation). The duration of the pulse exposure to CN^−^ (60 s) was selected according to the work by Hanawa et al. [37]. The concentrations of CN^−^ initially selected for the pulse exposure to this poison in the present study were also those used by Hanawa et al. [37] (25.0 and 50.0 mg L^−1^). However, since preliminary experiments revealed that at 25.0 mg L^−1^ of CN^−^ some fish mortality occurred after an exposure of 60 s, it was decided to select 12.5 and 25.0 mg L^−1^ (rather than 25.0 and 50.0 mg L^−1^) of CN^−^ as the experimental concentrations to be tested during pulse exposure.

Two different chemicals are commonly employed in cyanide fishing: sodium cyanide (NaCN) and potassium cyanide (KCN). As both chemicals are potent poisons, it has been assumed that their fish-stunning capacity does not differ significantly [38]. In order to allow a better comparison of experimental results, the present work selected the same chemical as Hanawa et al. [37] - NaCN. A stock solution of 18.84 g L^−1^ of NaCN (97% purity; Sigma-Aldrich, St. Louis, MO, USA), with a CN^−^ concentration of 10.00 g L^−1^, was prepared by dissolving 5.65 g NaCN in 300 mL deionized (Milli Q) water. The experimental concentrations of CN^−^ employed during the pulse exposure (12.5 and 25.0 mg L^−1^) were prepared by adding 18.7 mL and 37.5 mL of the stock solution to 15 L of synthetic seawater, respectively.

Following the pulse exposure to CN^−^, all fish were randomly distributed into 2-L glass jars (0.12 m in diameter and 0.25 m tall) filled with 1.5 L of synthetic seawater for post-exposure depuration (see Figure 1 for a schematic representation). All glass jars were equipped with an air stone to provide gentle water aeration. The jars were placed inside a water bath keeping water temperatures at 26±0.5°C and exposed to a photoperiod of 12 h light:12 h dark provided by white fluorescent lamps. The behavior of all fish was monitored during the first 30 minutes inside the glass jars to record recovery and mortality promoted by cyanide pulse exposure. The clownfish were allowed to depurate for a 4 week period, as this time-window overlaps with the period commonly elapsing between the collection of a marine ornamental fish and its purchase in retail stores by hobbyist [37]. During the depuration period, the water from each jar was fully replaced every day after feeding the fish to satiation with the commercial pelleted feed. The objective of this procedure was to avoid any degradation of water quality promoted by decaying uneaten food. A water sample of 1 mL was collected daily with a micropipette from each jar prior to feeding and stored at −20°C for determination of SCN^−^ concentrations the following day. Each time a new batch of synthetic seawater was prepared (including the seawater used to prepare the CN^−^ solution for pulse exposure, as well as cleaning baths) water samples were collected in order to determine any background levels of SCN^−^.

Time-zero measurements of SCN^−^ levels were performed in the 2-L glass jars employed to hold the fish in order to ensure that no false positives were recorded. Additionally, a preliminary study confirmed that no retention of SCN^−^ occurred in the 2-L glass jars after repeated exposure to this compound over a period of 4 weeks (the same duration as the depuration period employed in our study).

### Thiocyanate analysis

The concentration of SCN*^−^* present in the water samples collected during the experimental period were determined using a new methodology based on optical fiber (OF) detection coupled to a liquid chromatography system (see File S1) [34]. Standards of SCN^−^ (4, 50, 100, 200, 300 and 400 µg L-1) and seawater samples (20 µL collected from each 2-L jar employed for stocking fish during the depuration period) were introduced by a micro-syringe (Hamilton, Bonaduz, GR, Switzerland) at the top of the injector unit of our OF apparatus. After separation on the column the analytes reached the analytical tube containing the coated OF sensor to generate an analytical signal. Calibration models were built by injecting 20 µL of different concentrations (4, 50, 100, 200, 300 and 400 µg L-1) of standard solutions. The concentration of SCN^−^ was determined by direct interpolation into the calibration curve within the linear dynamic range, with detection limits being calculated by using the following formula: y = yB+3 sB, where sB is the standard deviation (SD) of the blank signal estimated as sy/x, the residual SD taken from the calibration line, and yB is the blank signal estimated from the intercept also taken from the calibration line. The accuracy recorded for our OF methodology was high, as the error recorded ((found value - expected value)/expected value)×100) was lower than 0.4%. This new technique based on OF detection coupled to a liquid chromatography system displays a high analytical performance, both in terms of linear range (4–400 µg L^−1^) and detection limits (3.16 µg L^−1^), an analytical time of less than 6 min, and is comparable to methodologies employing high performance liquid chromatography with UV detector (HPLC-UV) [34].

### Statistical analysis

The existence of significant differences in the levels of SCN*^−^* in the water samples collected during the experiment (28 days) were analyzed using a repeated measurements ANOVA, with the concentration of cyanide employed during the pulse exposure being used as the categorical factor. Statistical analyses were performed using the software STATISTICA version 8.0 (StatSoft Inc.), with the assumptions of normality and homogeneity of variance checked prior to analysis through the Shapiro-Wilks and Levene test, respectively. Mauchly's test of sphericity was used to determine if the variances of the differences between all combinations of related groups (levels) are equal. Whenever significance was accepted, at p<0.05, the Tukey multiple comparison test was used for pairwise comparison of means [39].

### Ethics Statement

This study was carried out in strict accordance with the recommendations present in the Guide for the Care and Use of Laboratory Animals of the European Union – in Portugal represented by the Decreto Lei n°129/92 de 06 de Julho, Portaria n°1005/92 de 23 de Outubro de 1992. Approval by a named review board institution or ethics committee was not necessary as the final model for ethical experimentation using fish as biological models was not implemented in Portuguese research units at the time of experimentation. This work was conducted under an institutional license for animal experimentation and a personal license to first author Marcela Vaz, and to the co-authors Isabel Lopes and Ruth Pereira, issued by the Direcção Geral de Veterinária (DGV), Portuguese Ministry of Agriculture, Rural Development and Fisheries.

## Results

### Fish anesthesia and mortality following pulse exposure to cyanide

During the pulse exposure to CN^−^ at both tested concentrations all fish displayed frantic swimming and strong gasping behavior, followed by a loss of balance, cessation of swimming (specimens rested motionless at the bottom of the container), and finally a complete loss of respiratory activity after about 30 and 50 s of the beginning of the pulse exposure with 12.5 and 25.0 mg L^−1^ of CN^−^, respectively. One and two specimens, respectively, of *A. clarkii* exposed to 12.5 and 25.0 mg L^−1^ of CN^−^ did not recover from anesthesia and died within 30 minutes following pulse exposure. Apart from a more agitated swimming behavior due to the netting during the 3 steps of the pulse exposure, fish in the control group did not display any of these responses and no mortality was recorded.

### Fish mortality and thiocyanate excretion during depuration

A single specimen of *A. clarkii* died during the depuration period at day 18 post-exposure (a fish previously exposed to 25.0 mg L^−1^ of CN^−^). The total number of *A. clarkii* reaching the end of the depuration process (28 days) were 9, 8 and 6 specimens, respectively, for the control group, and the groups pulse-exposed to 12.5 mg L^−1^ or 25.0 mg L^−1^ of CN^−^.

**Figure 2:**
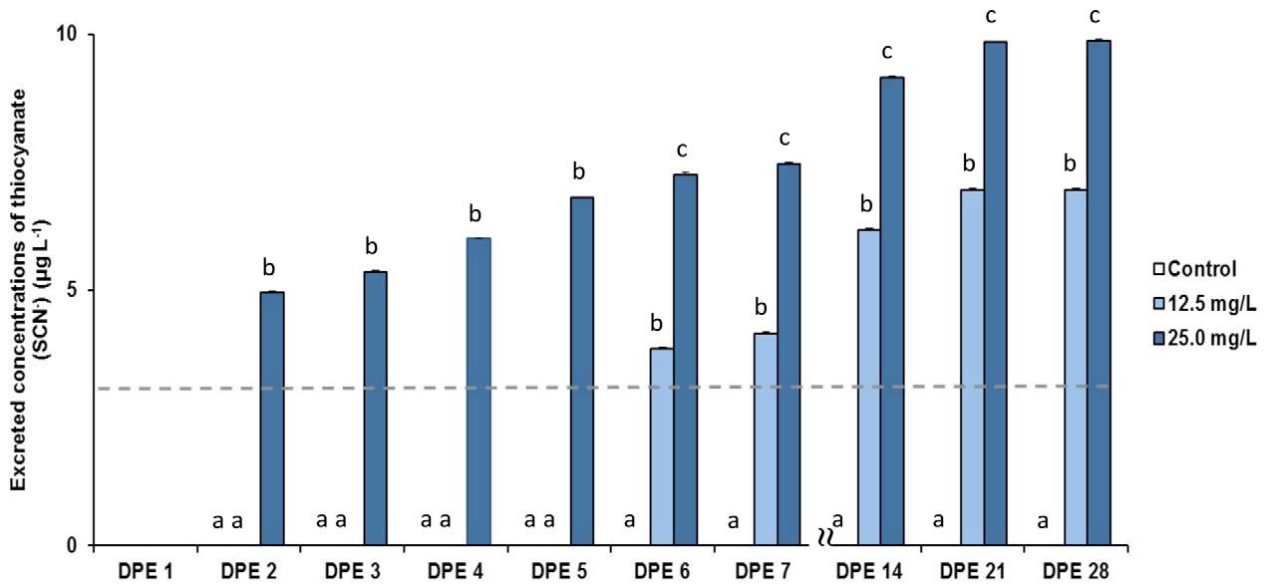
Concentrations (µg L^−1^) of thiocyanate (SCN^−^) excreted during the depuration period of *Amphiprion clarkii*. Values are averages ± standard error of SCN^−^ excreted by fish not exposed to cyanide (control group) or pulse exposed to 12.5 or 25.0 mg L^−1^ of cyanide. The grey dashed line represents the detection limit (3.16 µg L^−1^) of the optical fiber methodology employed to determine SCN^−^concentrations on the water samples collected from the jars holding *A. clarkii* during the depuration period. Different superscript letters in columns in the same day post-exposure (DPE) represent significant differences at p<0.05.

During the whole depuration process no detectable levels (>3.16 µg L^−1^) of SCN*^−^* were recorded in the water samples collected from the jars holding fish from the control group (no exposure to CN^−^) (Figure 2). On the first day post-exposure (DPE), no detectable levels of SCN*^−^* were recorded for water samples collected from jars holding fish pulse-exposed to CN^−^. However, on the second DPE an average concentration (± SE) of 4.95±0.04 µg L^−1^ of SCN*^−^* was already detected in the water holding *A. clarkii* pulse-exposed to 25.0 mg L^−1^ of CN^−^. Concerning *A. clarkii* pulse-exposed to 12.5 mg L^−1^ of CN^−^, SCN*^−^* was only detected 6 days after exposure (3.84±0.04 µg L^−1^). At that time, water samples collected from specimens pulse-exposed to 25.0 mg L^−1^ of CN^−^ displayed a significantly higher content of SCN*^−^* (7.25±0.05 µg L^−1^) (p<0.0001) (Figure 2). By the end of the third depuration week (21 DPE), SCN*^−^* levels in the water had increased to 6.96±0.03 and 9.84±0.03 µg L^−1^, respectively, for fish pulse exposed to 12.5 and 25.0 mg L^−1^ of CN^−^, and remained significantly higher in specimens pulse-exposed to a higher concentration of CN^−^ (p<0.0001). When the experiment ended (28 DPE), SCN*^−^* levels in the water holding fish pulse-exposed to 12.5 and 25.0 mg L^−1^ of CN^−^ remained nearly identical to those recorded at 21 DPE (Figure 2). No detectable levels of SCN*^−^* (>3.16 µg L^−1^) were recorded in the samples collected from the batches of newly prepared synthetic seawater.

## Discussion

The present work confirms that our OF methodology can be used to determine elevated concentrations of SCN^−^ in seawater samples collected from containers holding fish previously pulse-exposed to CN^−^.This non-invasive and non-destructive approach, with an analytical time of less than 6 min [34], allowed us to monitor during a 4 week period the daily excretion of SCN^−^ by fish previously exposed to CN^−^. The data demonstrate how SCN^−^ excretion varied according to the initial concentration of CN^−^ during pulse exposure, as well as how long SCN^−^ was detectable after water samples were collected for analysis. While detectable levels of SCN^−^ started to be excreted by fish the day following pulse-exposure to 25.0 mg L^−1^ of CN^−^, it took nearly one week for fish that had been exposed to a lower concentration of CN^−^ (12.5 mg L^−1^) to start excreting detectable levels of SCN^−^. Other aspects that may influence the excretion of SCN^−^ by fish exposed to cyanide are: 1) the duration of the exposure period, 2) fish species and size, 3) collection and post-collection handling stress, 4) the maintenance time and husbandry practices of exporting or importing facilities, and 5) shipping duration [18], [23], [37]. Although there is some available information on the initial concentrations of CN^−^ on apprehended squirt bottles employed for cyanide fishing (ranging from 760 to more than 2000 mg L^−1^) [40], the absolute concentrations to which fish are exposed in the field are still unknown. Consequently, it is nearly impossible to predict how soon marine fishes collected with cyanide will start to excrete SCN^−^ in detectable levels by our FO methodology.

Our work may also indicate that fish species and size can influence the effects of CN^−^ pulse exposure, as a concentration and exposure time that promoted no mortality in *Dascyllus aruanus* (25.0 mg L^−1^ of CN^−^ and 60 s) [37] was lethal for some of the *A. clarkii* specimens with slightly smaller size in the present study. If such physiological variability can occur among members of the same family (both *D. aruanus* and *A. clarkii* belong to the Pomacentridae [41]), interspecific variation is expected in the post-exposure metabolism of CN^−^ and depuration kinetics of SCN^−^, as at least 36 different reef fish families have already tested positive for cyanide fishing [18]. Our study has not allowed us to determine for how long fish exposed to CN^−^ continue to excrete SCN^−^ in detectable levels, as depuration was interrupted 28 DPE. However, our data agree with the findings by Rubec et al. [18] on the prevalence of CN^−^ in marine fish tissues for up to 3 weeks after cyanide fishing. Our study also supports the assertions of Rubec et al. [33] that marine fish do not quickly convert CN^−^ into SCN^−^, and that SCN^−^ is not excreted through the urine of marine fish in a matter of hours following cyanide exposure. It appears likely that this occurs because the low rate of urinary excretion of SCN^−^ by marine fish promotes the retention of SCN^−^ for longer time periods [33].

By increasing the holding period in export facilities of fishes beyond the end-point of SCN^−^ excretion, traders may avoid detection by our OF methodology. However, when trading cyanide-caught fishes, it is a common practice along the chain-of-custody to sell these specimens as fast as possible, in order that losses occur in the next link of the chain (ultimately the marine aquarium hobbyist). By keeping cyanide-caught fish for longer periods in their facilities wholesalers and/or retailers also risk losing fish due to their high mortality [8]. Additionally, they could also be subject to prosecution, since law enforcement officials are more likely to detect SCN^−^ in seawater holding tanks as cyanide-caught fish would be held during the excretion period of SCN^−^.

Traders acting in good faith and willing to avoid potential false positives should export their live reef fish in synthetic seawater and not in natural seawater. The main reason for employing this practice is that natural seawater can display SCN^−^ levels higher [34] than those recorded in the present study. Enforcement personnel in importing countries can avoid recording false positives simply by isolating the fish to be tested for 24 h in a container with artificial seawater (e.g., during the quarantine period) and only then employing the OF methodology to detect excreted SCN^−^.

In conclusion, our OF methodology: 1) tests for the compound that is excreted in the urine by fishes poisoned with cyanide (SCN^−^, rather than CN^−^); 2) delivers results much faster than current techniques employed for testing total CN^−^, which are time-consuming and labor intensive; 3) is non-invasive and non-destructive (no need for taking fish muscle or blood samples), and does not require the sacrifice or a heavy manipulation of the fish; and 4) is easier, safer and cheaper to use than current techniques available to detect live reef fish collected illegally using cyanide. This approach can be used to screen live reef fish immediately upon arrival to importing countries. In importing countries it is easier to take legal action against traders landing cyanide-caught fish. Authorities of importing countries can also provide information to the exporting countries regarding which exporters supply fish illegally collected with cyanide. This strategy may discourage importing enterprises from dealing with unreliable suppliers and shift the legal pressure from impoverished fishermen to those truly profiting from this illegal and destructive activity.

## Supporting Information

File S1 **PDF file of the manuscript describing in detail the methodology employed in this work to determine thiocyanate (SCN^−^) in seawater.**
(PDF)
