# Supplementary material for: Excreted Thiocyanate Detects Live Reef Fishes Illegally Collected Using Cyanide—A Non-Invasive and Non-Destructive Testing Approach
Source: PLoS One. 2012 Apr 20;7(4):e35355. doi: 10.1371/journal.pone.0035355 (PMC3335052; doi:10.1371/journal.pone.0035355)
Supplement: File S1 — PDF file of the manuscript describing in detail the methodology employed in this work to determine thiocyanate (SCN−) in seawater. (PDF) [file pone.0035355.s001.pdf]

Journal of Environmental Monitoring

Cite this: *J. Environ. Monit.*, 2011, **13**, 1811

www.rsc.org/jem

View Online

Dynamic Article Links

PAPER

Optical fiber based methodology for assessment of thiocyanate in seawater

Lurdes I. B. Silva,*[a] Celine I. L. Justino,[a] Isabel Lopes,[b] Ruth Pereira,[b] Ana Cristina Freitas,[c] Ricardo Calado,[b] Teresa A. P. Rocha-Santos,[ac] Teresa S. L. Panteleitchouk,[c] Maria E. Pereira[a] and Armando Costa Duarte[a]

Received 19th January 2011, Accepted 21st March 2011
DOI: 10.1039/c1em10043b

Downloaded by Universidade de Aveiro (UAveiro) on 06 July 2011
Published on 15 April 2011 on http://pubs.rsc.org | doi:10.1039/C1EM10043B

A new methodology for the assessment of thiocyanate (SCN^-) is proposed based on optical fiber (OF) detection coupled to a liquid chromatography system (LC). The developed methodology showed an adequate performance for the analysis of SCN^- comparable to a high performance liquid chromatography with UV detector (HPLC-UV) methodology: a detection limit of 3 μg L^{-1}, a linear range from 4 to 400 μg L^{-1}, and an analytical time of less than 6 min. The OF based methodology was of compact design and easy operation. This simple system has the potential to be used as a sensing approach for SCN^- in seawater.

Introduction

Thiocyanate (SCN^-) has been widely used in fabric dyeing, photography and electroplating industry,[1] and it has remarkable hazardous effects on both the environment and human health, as it is known to block the iodine uptake by the thyroid gland.[1,2] The accurate determination of SCN^- levels in marine water can also be of paramount importance for the detection of live coral reef fishes collected using an illegal and highly destructive technique—cyanide fishing.[3,4] Live fishes traded for human consumption or marine aquariums which have been exposed to cyanide are known to naturally excrete SCN^- as a way of self-depuration.[5] If SCN^- levels recorded in the water holding recently imported fishes exceed the basal values recorded for seawater, this can be considered as evidence that imported fishes were actively excreting SCN^- and have been illegally collected with cyanide.

Several analytical methods for the determination of SCN^- in various types of samples have been previously reported elsewhere, with molecular spectroscopic methods (including UV-Vis spectrophotometry and spectrofluorimetry) being the most widely applied.[1,6,7] The most used spectrophotometric method for thiocyanate determination is based on the formation of a red complex ($\lambda = 480$ nm) with ferric ion, $Fe(SCN)^{2+}$, in acid medium.[1]

Other methods used for determination of SCN^- are electrochemical methods (with ion selective electrodes),[8] gas chromatography with electron capture,[9] capillary electrophoresis,[10] sensors based on fluorescence detection,[11] and liquid chromatography coupled to an UV detector (LC-UV).[12] Most of these methods either require sophisticated operation or take long operation times. Additionally, all the methods referred, with exception of LC-UV, cannot be directly applied to the determination of thiocyanate in seawater. Therefore, there is still need for the development of a simple, fast and reliable method for determination of SCN^- in seawater.

Optical fiber (OF) sensors and OF based methodologies have been proven to be of high sensitivity and of easy operation for several analytes.[13–26] In OF sensors based on polymeric film as

[a]*CESAM & Department of Chemistry, University of Aveiro, Campus Universitário de Santiago, 3810-193 Aveiro, Portugal. E-mail: lisilva@ua.pt; Fax: +351 23437084; Tel: +351 234370200*
[b]*CESAM & Department of Biology, University of Aveiro, 3810-193 Aveiro, Portugal. Fax: +351 234372587; Tel: +351 234370788*
[c]*ISEIT/Viseu—Instituto Piaget, Estrada do Alto do Gaio, Galifonge, 3515-776 Lordosa, Viseu, Portugal. E-mail: lsilva@viseu.ipiaget.org; Fax: +351 232910183; Tel: +351 232910100*

Environmental impact

Thiocyanate (SCN^-) has remarkable hazardous effects on both the environment and human health, as it is known to block the iodine uptake by the thyroid gland. Fishes exposed to cyanide, a highly destructive technique used for coral reef's fish collection, are known to naturally excrete thiocyanate as a way of self-depuration; these organisms are traded live and present a high market value both for human consumption and/or the marine aquarium trade. Therefore, obtaining reliable data on SCN^- concentration in seawater becomes an important aim both in environmental sciences and sensing technology. An analytical methodology based on optical fiber detection has been developed for thiocyanate assessment in seawater. This methodology is capable of inexpensive, fast and direct analysis of thiocyanate in seawater samples and therefore could be applied to non-invasive and non-destructive detection of coral reef fishes collected using cyanide fishing.

View Online

Downloaded by Universidade de Aveiro (UAveiro) on 06 July 2011
Published on 15 April 2011 on http://pubs.rsc.org | doi:10.1039/C1EM10043B

the sensitive material/component, the analytical signal generation and characteristics must be understood as an interaction between several physical and chemical factors. Firstly, the analytical signal depends on the wave guide, the polymeric cladding and the analyte properties. Secondly, it is important to take into account the molecular interactions that occur between the analyte molecules and the polymeric film with consequent changes in the intensity of the light power guided through the OF.[27,28] When the analyte molecules contact with the polymeric film, the optical power intensity will vary proportionally to the amount of the analyte present. Without losing sight of the advantages of OF sensors, the aim of this work was to develop an OF based methodology for detection of thiocyanate in seawater samples. The developed methodology was validated by comparison with a high performance liquid chromatography with UV detector (HPLC-UV) methodology and applied to the determination of thiocyanate in seawater samples.

Experimental

Preparation of calibrants for thiocyanate determination

Standard solutions of SCN^- (4, 50, 100, 200, 300 and 400 µg L^{-1}) have been prepared by dilution of NaSCN, (Sigma, Sintra, Portugal) in artificial seawater. Artificial seawater was prepared using freshwater purified by a reverse osmosis unit and mixed with the synthetic salt Crystal Sea® produced by Marine Enterprises International® (Baltimore, MD, USA), following the instructions of the manufacturer.

Preparation of standard solutions for comparison of methodologies

In order to test and compare the performance of the proposed LC-OF methodology with HPLC-UV, ten different concentrations of SCN^- (10, 70, 100, 130, 190, 220, 250, 310 and 370 µg L^{-1}) have been prepared, and determined with both methodologies performing five repeated evaluations for each concentration tested.

OF based methodology

The schematic representation of the experimental apparatus of OF based methodology is shown in Fig. 1a. The injector unit (I) was connected to the pump (P) which was connected to the mobile phase flask (MP). The column (C) (150 mm × 4.6 mm i.d., C30 column modified with 5% polyethylene glycol (PEG) 20 000) was connected to the injector unit (I) and to the analytical tube (AT). The AT had an internal narrowed region of 0.4 cm diameter and 6.5 cm long having inside the OF that is a monomode optical fiber pigtail, core and cladding diameters of 9 and 125 µm, respectively, integrated into a directional 50:50 Y optical coupler (OC), with an angle FC/PC connector and a super FC/PC on the input ends. The optical fiber was previously uncladded, cleaved to 15 mm of the optical path cord and dipped (the cleaved section) into a PEG solution, by dip-coating technique, resulting in the sensitive component of the OF detection. Tests concerning the stability/durability of the sensitive OF section by optical signal monitoring during 3 months and SEM observation of the sensitized OF section revealed that no significant signal degradation and morphology changes were recorded, respectively. Additionally, the sensitive component (uncladded OF section + polymeric film) of the OF methodology can be replaced, after 3 months of continuous operation, in order to maintain high analytical performance after extensive utilization for longer periods of the analytical sensing system. Besides the coated optical fiber and the analytical tube, the detection component of the OF based methodology was constituted by a laser diode optical source (L, Oz Optics, Ottawa, Canada) and a photodiode detector (PD, Oz Optics, Ottawa, Canada). The laser diode (1 mW) optical source was (a) set at 1550 nm for working wavelength and at continuous waveform (CW) regarding the operational mode frequency, to generate the interrogating signal, and (b) connected to the optical coupler (OC). The OC was also connected to the photodiode detector (P), which measures the intensity of the modulated signal, and to a laptop (PC) with homemade software.

Standards of SCN^- (4, 50, 100, 200, 300 and 400 µg L^{-1}) and seawater samples (20 µL) were introduced by a micro-syringe (Hamilton, Bonaduz, GR, Switzerland) at the top of the injector (I). After separation on the column the analytes reached the analytical tube (AT), which contains the coated OF, generating an analytical signal (Fig. 1b). The changes in the reflected optical power caused by variations in the OF refractive index have been previously discussed by Silva *et al.*,[13–23] and depend on the coating film (PEG) and analyte properties, as well as the chemical interactions which can take place between these two elements. The mobile phase was 300 mM sodium sulfate and 50 mM sodium chloride and the flow-rate was 1.0 mL min^{-1}.

Calibration models were built by injection of 20 µL of different concentrations (4, 50, 100, 200, 300 and 400 µg L^{-1}) of standard solutions. The concentration of thiocyanate was determined by direct interpolation in the calibration curve within their linear dynamic range, and the detection limits were calculated using $y = y_B + 3s_B$, where s_B is the standard deviation (SD) of the blank signal estimated as $s_{y/x}$, the residual SD taken from the calibration line, and y_B is the blank signal estimated from the intercept taken also from the calibration line.[29]

HPLC-UV based methodology

An HPLC-UV based methodology[12] was used in order to test and compare the performance of the OF methodology. The column used was C30 column (150 mm × 4.6 mm i.d.) modified with 5% PEG 20 000. The mobile phase was 300 mM sodium sulfate and 50 mM sodium chloride and the flow-rate was 1.0 mL min^{-1}. Standards of thiocyanate were injected (20 µL) on a HPLC-UV (Jasco PU980, Easton, USA) and the wavelength of UV detection was 220 nm.

Sampling of thiocyanate

Seawater samples were collected in clear screw neck glass vials (Fisherbrand, Fisher Scientific, UK) during the months of June and July 2010 from several locations off Aveiro in the Portuguese Coast (Fig. 2). Collected samples were filtered through a 0.45 mm membrane filter and kept under refrigeration at 4 °C before further analytical processing.

This journal is © The Royal Society of Chemistry 2011

View Online

Downloaded by Universidade de Aveiro (UAveiro) on 06 July 2011
Published on 15 April 2011 on http://pubs.rsc.org | doi:10.1039/C1EM10043B

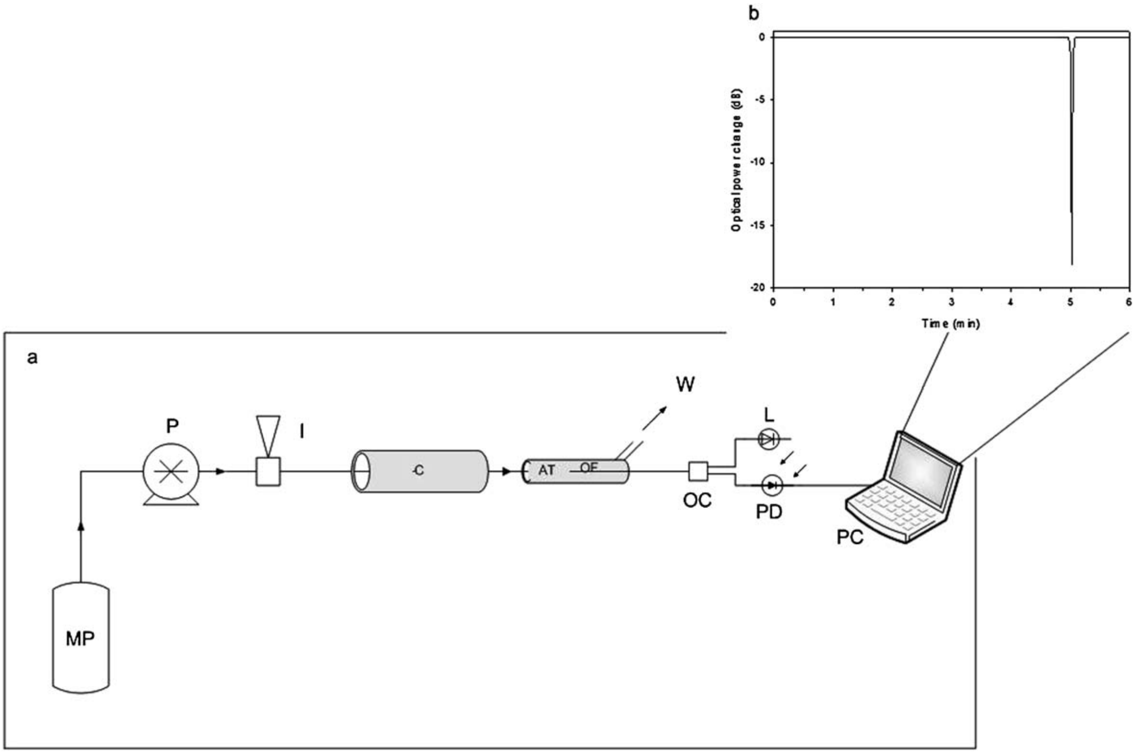

Fig. 1 (a) Schematic representation of the experimental apparatus used for OF based methodology (MP = mobile phase flask; P = pump; I = injector; C = column; AT = analytical tube; OF = optical fiber; W = waste; OC = optical coupler; L = laser diode optical source; PD = photodiode detector; PC = laptop with homemade software); (b) analytical signal obtained by OF based methodology for a standard of thiocyanate.

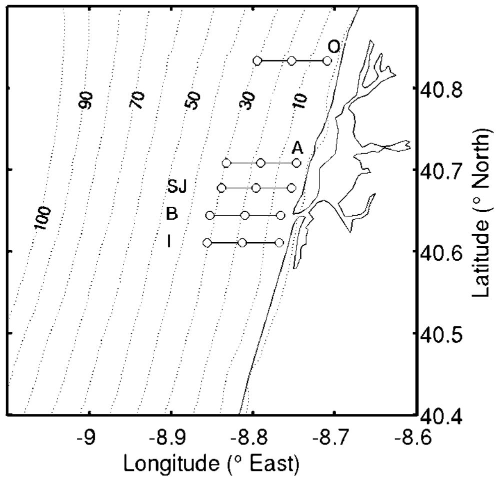

Fig. 2 Study area and sampling locations off Aveiro in the Portuguese Coast. A—Aveiro, B—Barra, I—Ilhavo, O—Ovar and SJ—São Jacinto.

Results and discussion

Table 1 summarizes the main analytical characteristics obtained for thiocyanate with the two tested methodologies. The analytical time (less than 6 min) was the same for both approaches, since the same chromatographic components and operational conditions (such as the mobile phase) were used.

In terms of retention time, linear range and detection limit, the results achieved were in the same order of magnitude for HPLC-UV and OF based methodologies. Both approaches displayed a retention time of approximately 5 min, a linear range from 4 to 400 µg L^{-1}, while the detection limits were 4 µg $L^{-1}$and 3 µg L^{-1} for the HPLC-UV and OF based methodologies, respectively.

From the calibration study, it was also found that the analytical performance of the OF based methodology reaches a plateau in terms of maximum capacity of detection for concentration values much over the linear range,[30] particularly for concentrations higher than 500 µg L^{-1}.

Ten standards with different concentrations of thiocyanate were used to evaluate the performance of the OF methodology *versus* the HPLC-UV methodology. The statistical analysis[31] of the obtained results suggests: (a) that a linear correlation can be established between the two analytical methodologies for the analysis of thiocyanate, with squared correlation coefficient R^2 of 1 ($p < 1.59 \times 10^{-33}$); (b) that the results obtained with the two analytical methodologies cannot be statistically differentiated since the regression line has an intercept and a slope not significantly different from 0 ($p = 0.944$) and 1 ($p < 1.59 \times 10^{-33}$), respectively; and finally (c) that there is a low dispersion level of the results obtained by the two applied analytical methodologies for thiocyanate analysis supported by an extremely narrow interval at a 95% confidence level.

The error, that is ((found value − expected value)/expected value) × 100) was lower than 0.4% for the OF methodology, and lower than 0.3% for the HPLC-UV methodology.

Apart from keeping the quality of the analysis of SCN^- at the same pattern of other usual methods of analysis, *i.e.* HPLC-UV, the developed methodology compares advantageously due to: (a)

View Online

Table 1 Analytical parameters obtained for thiocyanate with HPLC-UV and OF based methodologies

	HPLC-UV	OF
Retention time/min	5.14	5.02
Linear range/μg L^{-1}	4–400	4–400
Linear calibration	$y = 3.52 + 0.147x$	$y = 2.77 + 0.073x$
Squared coefficient of correlation/R^2	0.9999 ($p < 6.33 \times 10^{-9}$)	0.9999 ($p < 3.57 \times 10^{-9}$)
Detection limit/μg L^{-1}	4	3

Downloaded by Universidade de Aveiro (UAveiro) on 06 July 2011
Published on 15 April 2011 on http://pubs.rsc.org | doi:10.1039/C1EM10043B

easiness of operation and effective cost of equipment, (b) compact and versatile design of the analytical system, (c) possibility of remote data acquisition, and (d) high potential for miniaturization, allowing the possibility of *in situ* analysis.

The compact design (Fig. 1) of the OF based methodology allows the integration of the system components into a portable structure for field application, *i.e.*, for thiocyanate *in situ* analysis. This structure should include a sampling tube and a mini-valve, automatically controlled by software and connected to the pump (P, Fig. 1). An electronic platform/software should be also implemented in order to (a) provide direct readings of the analytical signal or (b) database for measurements storage, and (c) remote data acquisition by integration in a wireless network.

After development, comparison and validation against HPLC-UV methodology, the OF methodology was applied to the analysis of thiocyanate in 15 seawater samples collected off Aveiro in the Portuguese Coast. The results from this analysis are summarized in Table 2.

In Table 2, it can be observed that the higher values of thiocyanate were found near the coast (1 km) decreasing to below the detection limit with increasing the distance to the coast. This fact could be related to anthropogenic activities since at off-shore there are larger water bodies which decrease this effect resulting in a decrease of SCN^- levels.

Moreover, the levels of thiocyanate found in the water samples collected on the Portuguese coast were in the same range of levels found for seawater samples collected on the Japanese coast (8.1–15.0 μg L^{-1}) by Rong *et al.*[12]

Table 2 Concentration of thiocyanate in seawater samples collected off Aveiro in the Portuguese Coast (mean and standard deviation obtained for five experiments)

Sampling site	Distance to the Coast/km	Thiocyanate/μg L^{-1}
Aveiro	2	20.5 ± 0.3
Aveiro	17	<3.3
Aveiro	24	<3.3
Barra	1	21.3 ± 0.3
Barra	5	7.2 ± 0.1
Barra	8	<3.3
Ilhavo	1	22.7 ± 0.3
Ilhavo	5	<3.3
Ilhavo	9	<3.3
Ovar	1	18.2 ± 0.2
Ovar	7	<3.3
Ovar	26	<3.3
São Jacinto	1	16.0 ± 0.2
São Jacinto	4	5.1 ± 0.1
São Jacinto	8	<3.3

Taking into account the complexity of seawater, and in order to access the applicability of the OF based methodology for the analysis of SCN^- in this matrix, the potential interference of iodide (concentration of 90 μg L^{-1}) in thiocyanate detection was evaluated. No interference could be detected, as iodide produces a peak in a different time window (around 2 min) of the target analyte (SCN^-), as shown in Fig. 3. Moreover, interferences from other analytes such as iodate were also evaluated and no interference was observable since the peak produced was also in a different time window (at 1.20 min).

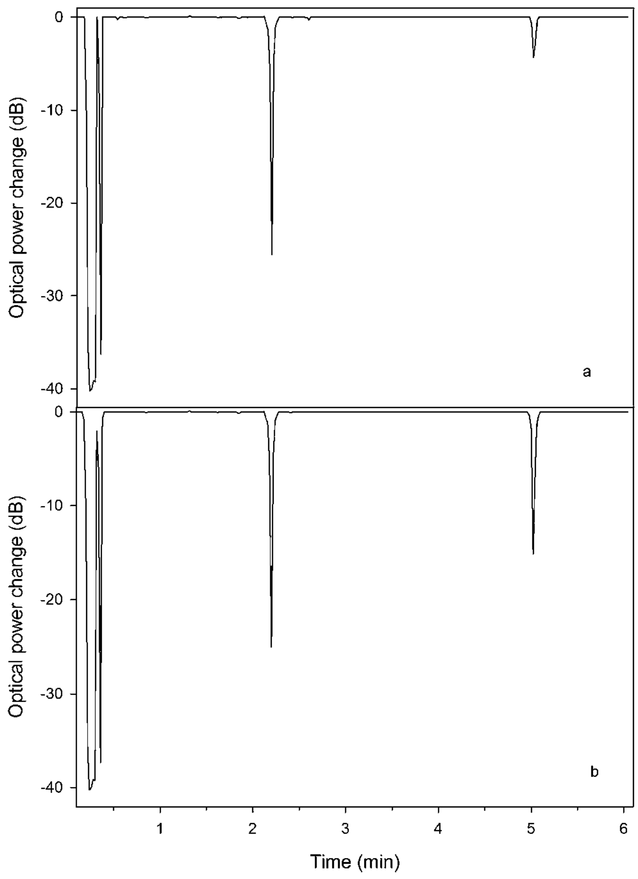

Fig. 3 Analytical signal obtained by OF based methodology (a) for a sample of seawater from Barra (off Aveiro in the Portuguese Coast) with a distance to the coast of 1 km, and (b) for a sample of seawater from Barra with a distance to coast of 1 km with thiocyanate standard addition. Iodine is the peak at 2.20 min and thiocyanate is at 5.02 min.

 This journal is © The Royal Society of Chemistry 2011

View Online

Downloaded by Universidade de Aveiro (UAveiro) on 06 July 2011
Published on 15 April 2011 on http://pubs.rsc.org | doi:10.1039/C1EM10043B

Conclusions

The OF methodology showed a high analytical performance, mainly in terms of linear range (4–400 μg L^{-1}) and detection limits (3 μg L^{-1}). It has proven to be adequate for the assessment of thiocyanate in seawater and comparable to HPLC-UV methodology. Simple and fast analysis of thiocyanate in seawater, easiness of use and compact design were also analytical features of the developed OF based methodology. In this way, this new methodology can constitute a platform for inexpensive analysis of thiocyanate in environmental samples without iodide interferences. A good example for a practical application of this new methodology is the non-invasive and non-destructive detection of highly priced coral reef fishes collected with cyanide (by rapidly and accurately tracing abnormally high levels of SCN^- in the water holding these organisms, rather than sacrificing fish to detect cyanide levels in their tissues and/or blood).

Acknowledgements

This work has been developed under the scope of the Portuguese Science Foundation (FCT, Portugal) research grants SFRH/BD/60429/2009, SFRH/BPD/48028/2008 and SFRH/BPD/65410/2009. We thank Bruna Silva for collecting the saltwater samples during one of the scientific cruise of project ISCAD (PTDC/MAR/65760/2006) coordinated by Jesus Dubert. We also thank João Lencart e Silva for providing the vure illustrating the study area and sampling locations. This research was partially financed by a starting grant of CESAM, University of Aveiro.

References

1 M. G. Valdés and M. E. Díaz-García, *Crit. Rev. Anal. Chem.*, 2004, **34**, 9–23.
2 C. Bjergegaard, P. Møller and H. Sørensen, *J. Chromatogr., A*, 1995, **717**, 409–414.
3 C. Wabnitz, M. Taylor, E. Green and T. Razak, *From Ocean to Aquarium*, UNEP-WCMC, Cambridge, UK, 2003.
4 K. K. W. Mak, Y. Hideshi and R. Reinhard, *Biosens. Bioelectron.*, 2005, **20**, 2581–2593.
5 A. W. Bruckner and G. Roberts, *Proceedings of the International Cyanide Detection Testing Workshop, NOAA Technical Memorandum NMFS-OPR-40*, Silver Spring, Florida, 2008.
6 B. Gong and G. Q. Gong, *Anal. Chim. Acta*, 1999, **394**, 171–175.
7 D. G. Themelis and P. D. Tzanavaras, *Anal. Chim. Acta*, 2002, **452**, 295–302.
8 M. K. Amini, A. Rafi, M. Ghaedi, M. H. Habibi and M. M. Zohory, *Microchem. J.*, 2003, **75**, 143–150.
9 S. Kage, T. Nakata and K. Kudo, *J. Chromatogr., B: Biomed. Sci. Appl.*, 1996, **675**, 27–32.
10 Z. Glatz, S. Novalova and H. Sterbova, *J. Chromatogr., A*, 2001, **916**, 273–277.
11 Y. Zhang, H. Wang and R. H. Yang, *Sensors*, 2007, **7**, 410–419.
12 L. Rong, L. W. Lim and T. Takeuchi, *Chromatographia*, 2005, **61**, 371–374.
13 L. I. B. Silva, T. A. P. Rocha-Santos and A. C. Duarte, *Anal. Sci.*, 2008, **24**, 963–966.
14 L. I. B. Silva, T. A. P. Rocha-Santos and A. C. Duarte, *Sens. Actuators, B*, 2008, **132**, 280–289.
15 L. I. B. Silva, T. A. P. Rocha-Santos and A. C. Duarte, *Talanta*, 2008, **76**, 395–399.
16 L. I. B. Silva, A. M. Costa, A. C. Freitas, T. A. P. Rocha-Santos and A. C. Duarte, *Int. J. Environ. Anal. Chem.*, 2009, **89**, 183–197.
17 L. I. B. Silva, A. V. Panteleitchouk, A. C. Freitas, T. A. P. Rocha-Santos and A. C. Duarte, *Anal. Methods*, 2009, **1**, 100–107.
18 L. I. B. Silva, F. D. P. Ferreira, A. C. Freitas, T. A. P. Rocha-Santos and A. C. Duarte, *Talanta*, 2009, **80**, 853–857.
19 L. I. B. Silva, T. A. P. Rocha-Santos and A. C. Duarte, *J. Environ. Monit.*, 2009, **11**, 852–857.
20 L. I. B. Silva, T. A. P. Rocha-Santos and A. C. Duarte, *Talanta*, 2009, **78**, 548–552.
21 L. I. B. Silva, F. D. P. Ferreira, A. C. Freitas, T. A. P. Rocha-Santos and A. C. Duarte, *Food Chem.*, 2010, **123**, 806–813.
22 L. I. B. Silva, A. C. Freitas, T. A. P. Rocha-Santos, M. E. Pereira and A. C. Duarte, *Talanta*, 2011, **83**, 1586–1594.
23 L. I. B. Silva, D. Rodrigues, A. C. Freitas, A. M. Gomes, T. A. P. Rocha-Santos, M. E. Pereira and A. C. Duarte, *Food Chem.*, 2011, **127**, 222–227.
24 K. Peters, *Smart Mater. Struct.*, 2011, **20**, 013002 (17p).
25 C. Elosua, I. R. Matias, C. Bariain and F. J. Arregui, *Sensors*, 2006, **6**, 1440–1465.
26 O. S. Wolfbeis, *Anal. Chem.*, 2008, **80**, 4269–4283.
27 A. Abdelghani, J. M. Chovelon, N. Jaffrezic-Renault, M. Lacroix, H. Gagnaire, C. Veillas, B. Berkova, M. Chomat and V. Matejec, *Sens. Actuators, B*, 1997, **44**, 495–498.
28 X. Wang, H. Zeng, L. Zhao and J.-M. Lin, *Talanta*, 2006, **70**, 160–168.
29 J. N. Miller and J. C. Miller, *Statistics and Chemometrics for Analytical Chemistry*, Pearson Prentice Hall, New York, 5th edn, 2005.
30 L. I. B. Silva, M. Freitas, T. A. P. Rocha-Santos and A. C. Duarte, *Talanta*, 2010, **82**, 1403–1411.
31 *SigmaStat 3.0 (Statistic Software for Windows)*, Jandel Scientific, Erkrath, Germany, 1994.
